# Identification of Two New Mechanisms That Regulate Fruit Growth by Cell Expansion in Tomato

**DOI:** 10.3389/fpls.2017.00988

**Published:** 2017-06-12

**Authors:** Constance Musseau, Daniel Just, Joana Jorly, Frédéric Gévaudant, Annick Moing, Christian Chevalier, Martine Lemaire-Chamley, Christophe Rothan, Lucie Fernandez

**Affiliations:** UMR 1332 BFP, Institut National de la Recherche Agronomique, University of BordeauxVillenave d’Ornon, France

**Keywords:** EMS, fruit, growth, morphology, mutant, phenotype, tissue, tomato

## Abstract

Key mechanisms controlling fruit weight and shape at the levels of meristem, ovary or very young fruit have already been identified using natural tomato diversity. We reasoned that new developmental modules prominent at later stages of fruit growth could be discovered by using new genetic and phenotypic diversity generated by saturated mutagenesis. Twelve fruit weight and tissue morphology mutants likely affected in late fruit growth were selected among thousands of fruit size and shape EMS mutants available in our tomato EMS mutant collection. Their thorough characterization at organ, tissue and cellular levels revealed two major clusters controlling fruit growth and tissue morphogenesis either through (i) the growth of all fruit tissues through isotropic cell expansion or (ii) only the growth of the pericarp through anisotropic cell expansion. These likely correspond to new cell expansion modules controlling fruit growth and tissue morphogenesis in tomato. Our study therefore opens the way for the identification of new gene regulatory networks controlling tomato fruit growth and morphology.

## Introduction

Plant domestication has resulted in profound phenotypic changes in fleshy fruit-bearing species including the increase in fruit yield, sensorial and nutritional quality and shelf-life. Even if domestication led to a drastic reduction of the nucleotide diversity (Doebley et al., 2006), mutations were continuously accumulated in a recent diversification phase following this syndrome, offering opportunities for new phenotypes to arise. As a result, in addition to fruit weight, other major fruit developmental traits have been selected, among which the fruit shape that displays a wide diversity in species such as tomato and pepper (Frary et al., 2000; Paran and van der Knaap, 2007; Rodríguez et al., 2011) and melon (Périn et al., 2002; Monforte et al., 2014).

This diversity has been further exploited in order to fulfill specific needs of fruits for fresh consumption (e.g., table grapes, fresh market tomatoes) and for processing and mechanical harvesting (e.g., wine grapes, processing tomatoes). This improvement highly impacted tissue morphology and cellular characteristics of the fruit. To date, tissue morphology has drawn much less attention than fruit weight or shape, probably because of its inherent complexity. Although tools enabling the comparative description of internal fruit morphology have been published (Rodríguez et al., 2010), the bulky nature of the fruit still requires destructive analyses to score these traits. As a consequence, very few large scale and detailed studies taking into account fruit tissue morphology, similar to those previously conducted on leaves (Chitwood et al., 2014, 2016), have been published.

Cultivated tomato (*Solanum lycopersicum* L.) stands as the model species for *Solanaceae* and for fleshy fruit biology, especially when investigating the ripening process (Klee and Giovannoni, 2011). It is also an appropriate model to analyze fruit weight and tissue morphology because of the large existing phenotypic diversity. Information have been collected in large databases collecting the phenotype of thousands of tomato varieties found all other the world as well as of wild tomato accessions (TGRC^1^; BreeDB^2^). In addition, considerable genomic data have been generated in the recent years, including the genome sequences of a large number of cultivated tomato varieties and of the wild relatives *S. pimpinellifolium* and *S. pennellii* (Tomato Genome Consortium, 2012; Aflitos et al., 2014; Bolger et al., 2014; Lin et al., 2014). A number of loci controlling fruit weight and shape have been mapped (Tanksley, 2004). Those selected through the domestication and subsequent improvement processes have been identified (Lin et al., 2014) and six genes underlying the major QTLs have been cloned. These include the *fw2.2* and *fw3.2* fruit weight QTLs (Frary et al., 2000; Zhang et al., 2012; Chakrabarti et al., 2013), the *locule-number* and *fasciated* locule number QTLs (Muños et al., 2011; Xu et al., 2015) and the *ovate* and *sun* fruit shape QTLs (Liu et al., 2002; van der Knaap et al., 2004; Xiao et al., 2008) that affect flower meristem, ovary or very early stages of fruit development. Remarkably, regardless of the wide diversity in fruit size and shape observed in cultivated tomato (e.g., as much as 3900 carpel number genotypes described in the BreeDB database), a limited set of allelic variants identified in six genes are involved, alone or in combination, in the major variations in fruit morphology in domesticated tomato (van der Knaap et al., 2014). Indeed, the alleles of the major genes SUN, OVATE, LC, and/or FAS individually explain up to 71% of the fruit shape variations in cultivated tomato (Rodríguez et al., 2011). Thus, there is a clear need for isolating new regulators of fruit morphology and, as a first step, to identify new fruit growth modules in tomato.

Of special interest are the variations affecting cell enlargement during the expansion stage of the fruit, which remain largely underexplored. Cell expansion contributes the most to the final size of the fruit (Lemaire-Chamley et al., 2005). It is also regulated differentially in the various tissues within the fruit and thereby likely plays a key role in fruit tissue morphology. One key process associated with the cell expansion phase in tomato is endoreduplication that results in the formation of polyploid nuclei. Rather than determining a defined cell size, endoreduplication in tomato offers a potentiality for further cell growth through the adjustment of the cytoplasmic volume with the nuclear DNA content, according to the karyoplasmic ratio theory (Cheniclet et al., 2005; Bourdon et al., 2012). A large set of data resulting from functional analyses of candidate genes controlling the mitotic cycle/endocycle transition highlighted the tight relationship between nuclear ploidy and cell size (see De Veylder et al., 2011; Chevalier et al., 2014 for reviews). However, because they are mostly limited to known candidate genes, reverse genetic strategies can hardly give new insights into poorly characterized developmental processes.

Thus the reduced genetic diversity in cultivated tomato that limits the identification of minor or “hidden” loci as well as the limitations of reverse genetics approaches hamper our comprehension of the late fruit growth modules in tomato. One way to overcome these limitations is to create *de novo* a wide genetic and phenotypic diversity. High genetic diversity can be obtained through EMS (ethyl methanesulfonate) mutagenesis that creates point mutations evenly distributed over the genome. We and others previously generated tomato mutant collections which displayed extensive fruit phenotypic diversity (Menda et al., 2004; Saito et al., 2011; Just et al., 2013) and further showed that a population of few thousand highly mutagenized mutant lines is sufficient to find at least one severe mutation in every single tomato gene (Garcia et al., 2016; Shirasawa et al., 2016). Here we further exploit this diversity to unravel developmental modules determining fruit weight and tissue morphology during the cell expansion phase of the fruit and get insights into the underlying mechanisms.

## Materials and Methods

### Plant Material and Culture

Fruit weight and tissue morphology tomato (*S. lycopersicum* L.) mutants were isolated from a highly mutagenized EMS mutant collection produced in the miniature cv. Micro-Tom as previously described (Just et al., 2013; Petit et al., 2014). In a first step, 35 mutant lines previously identified as fruit size and/or morphology mutants were phenotyped to confirm the observed phenotypic alterations (six plants/mutant line); this was done on M2 or M3 plants. In a second step, each selected mutant line was self-pollinated and M4–M8 plants were used for detailed phenotypic analysis. Mutant phenotypes are therefore considered as fixed because genome homozygosity is in the ∼93 to 99% range. Phenotyping was carried out during 4 years (2012–2015) on tomato plants grown year-round (3–4 cycles/year) in greenhouse in controlled conditions as described in Rothan et al. (2016). In order to take into account the fruit phenotypic plasticity in changing environmental conditions (mainly due to seasonal variations), the non-mutagenized Micro-Tom parental line, thereafter called WT, was cultivated side-by-side with the mutant lines and used as a reference. In addition, for all the lines, the first flower from each inflorescence was removed to take into account the high incidence of abnormal fruits produced by this flower in cv. Micro-Tom. A total of 39 different parameters thoroughly describing the plant development (3), yield components (2), organ (6), tissue morphology (11), cell morphology (8) and nucleus ploidy (9) were used for phenotyping the mutants (Supplementary Table 1). Ovary analyses were performed at anthesis (i.e., fully opened flower) before fruit set. Fruit analyses were performed at breaker stage at the onset of ripening. Breaker stage was defined as the first appearance of yellowish traces on the fruit, which takes place at about 30–35 DPA when the fruit has reached its final size. To take into account the likely influence of photoassimilate availability on fruit growth and tissue morphology, most parameters (Supplementary Table 1) were measured in standardized conditions. To this end, fruit load on the plant was reduced to six fruits distributed on two fruit trusses (controlled load) by flower pruning. In addition, to allow comparison between controlled and unrestricted fruit loads, several parameters (Supplementary Table 1) were also measured from plants in which fruit load was left free and allowed to reach up to 20 fruits per plant (unrestricted load). In addition to ovary and fruit phenotypes, plant traits with possible effect on fruit growth (fruit yield, vegetative-to-reproductive phase transition) were also considered.

### Determination of Fruit Tissue Morphology

Fruit tissue morphology features were determined from fresh fruit equatorial sections by scoring the proportion of each fruit tissue. Fruit equatorial sections were analyzed using the Tomato Analyser 3.0^®^ software (Rodríguez et al., 2010). Whole fruit, pericarp, radial pericarp, columella and locular tissue area measurements were done according to Sun et al. (2015) (Supplementary Figure 1A). Values given for the proportion of pericarp (%P), radial pericarp (%RP), locular tissue (%LT) and columella (%C) were relative to the whole fruit and were thus independent from the variations in fruit weight observed amongst the various mutants.

### Ovary and Fruit Histological Analyses

Fresh equatorial sections of ovaries (30 μm thickness) and breaker fruits (150 μm) were obtained using a vibration microtome (Microm HM 650 V, Thermo Scientific) prior to staining with 0.01% calcofluor or 0.05% toluidine blue (TB). Three to eleven ovaries or fruits were analyzed for each mutant line (Supplementary Table 1). Sections were then observed under an epifluorescence microscope (Zeiss Axiophot, Carl Zeiss) for calcofluor staining or with a stereomicroscope (Olympus SZX16, Olympus) for TB staining. Pericarp measurements were performed on the three layers (exocarp, mesocarp, and endocarp), excluding the vascular bundles.

Average equatorial ovary area (O_A), ovary wall thickness (OW_thick), pericarp thickness (P_thick) and number of pericarp cell layers (Cell_layer) were determined using these sections (Supplementary Figure 1B). Measurements (5 per trait) were done and averaged using the Image-Pro PLUS software (Media Cybernetics, Silver Spring, MD, United States).

These sections were also used to measure the cell area within the ovary wall and the fruit pericarp. Cell segmentation was performed using the CellSeT software (Pound et al., 2012) to optimize automatic cell counting. Area quantification was done using the Image-Pro PLUS software (Supplementary Figure 1C). For area quantification, 100 to 300 cells were observed per section to evaluate the maximum cell area (OWCell_max, PCell_max), the mean cell area (OWCell_mean, PCell_mean) and the average cell area of the 25% larger cells (OWCell_25, PCell_25). The same segmentations were used to evaluate cell shape of the 10 and 25% largest cells by scoring the *X*/*Y* cell ratio where (*X*) corresponds to the adaxial–abaxial and (*Y*) to the medio-longitudinal axes.

### Ploidy Analysis

Cell ploidy quantification was determined by flow cytometry (CyFlow Space, Partec) using pericarp tissue from the fruit equatorial region according to Cheniclet et al. (2005). The relative proportion of each nucleus population (4C to 256C) was calculated together with the Ploidy Index (PI) and the Endoreduplication Factor (EF). Both are commonly used to estimate the mean ploidy level and the average number of endoreduplication cycles, respectively (Bertin et al., 2009). The 2C peaks were not quantified because of their low level in Micro-Tom at breaker stage (L. Fernandez, pers. obs.).

### Statistical Analyses

Multivariate and univariate analyses were performed using BioStatFlow application implemented using R packages (v2.7.7 INRA^3^) in order to identify differences and relationships between mutants or traits.

A Volcano plot was used to visualize significant phenotypic variations for fruit weight and pericarp thickness corresponding to the initial criteria used for mutant screening. Data from the different experiments were plotted in a single Volcano plot to insure consistency between the different environmental conditions. Mean comparisons between mutants and the WT were performed using Student’s *t*-test and choosing the false discovery rate (FDR) cut-off of 0.05 (Benjamini and Hochberg, 1995).

Principal component analysis (PCA) was used to have a global view of the data. As some parameters were analyzed over different experiments, for consistency, for each experiment the mutant values were expressed as relative to the WT (ratio between the mean value of the mutant and the mean value of the WT). The data used for PCA corresponded to the average mutant/WT ratios standardized to unit variance.

Clustering of the mutants was performed using correlation coefficients calculated on 24 fruit traits measured in controlled fruit load conditions. Spearman rank correlations were chosen in order to capture non-linear relationships. The correlations between traits were calculated separately for each mutant cluster based on the same fruit parameters together with ovary parameters. Correlations were calculated on the average mutant/WT ratios for each trait and considered significant when *P*-value < 0.01. Network reconstruction was performed using BioStatFlow application and visualization was done using Cytoscape software v3.1 using spring embedded layout (Shannon et al., 2003).

## Results

### Screening Micro-Tom EMS Mutant Collection for Fruit Weight and Tissue Morphology Mutants

To investigate the induced variations in fruit weight and tissue morphology, we screened a tomato mutant collection previously generated in the miniature cultivar Micro-Tom by EMS mutagenesis. About 3500 M2 mutant lines had previously been phenotyped for over 150 phenotypic traits; within this collection the fruit shape and size diversity observed was found to be considerable (Just et al., 2013). For example, we observed in the collection 1253 multi-locular mutant families. Increased carpel number and fruit shape alterations are indeed of considerable interest to investigate variations in fruit weight and tissue morphology in cultivated tomato (van der Knaap et al., 2014) but may also hinder the existing relationships between different fruit traits (e.g., the link between pericarp cell ploidy and fruit size; Cheniclet et al., 2005). We therefore excluded fruit carpel number mutants and fruit shape mutants (e.g., round, flat, rectangular, ovoid or heart shaped fruits; Supplementary Figure 2) from the analysis. Likewise, as fruit growth patterns are likely modified in the parthenocarpic fruits which often lack locular tissue and in addition are very difficult to multiply (Serrani et al., 2007), strict parthenocarpic mutants were excluded from the analysis. However, mutants with a reduced number of seeds that could be easily maintained through sexual propagation were included in the study. At last, care was also taken to exclude mutants showing major variations in plant height and leaf attributes (number, size, shape, and color), since the carbon status of the plant can considerably impinge on fruit growth.

**FIGURE 1:**
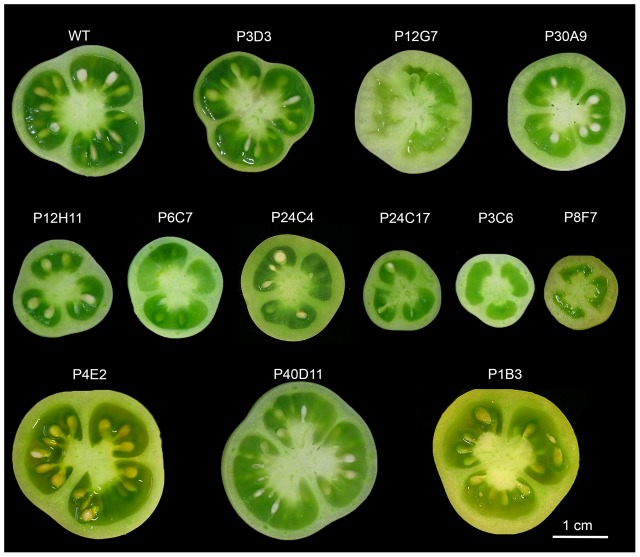
Diversity of fruit weight and tissue morphology of the 12 selected tomato mutants and the wild-type (WT). Fruit equatorial sections around breaker stage show small and large fruits phenotypes as well as thick- and thin-pericarp phenotypes. Locule number and fruit shape in the mutants are similar to the WT. No strict parthenocarpy phenotypes were selected and P12G7, P3C6 and P8F7 produced at least few seeds.

Phenotypic selection centered on fruit size and pericarp thickness produced, respectively, as many as ∼ 2300 and ∼1000 mutants in the collection. Taking into account the criteria indicated above, 35 mutants were selected and grown in a greenhouse. To investigate the developmental processes involved in tomato fruit growth, 12 mutants displaying robust and stable fruit phenotypes over the different environmental conditions experienced by the plants were then further characterized (**Figure 1**). All these mutants were comparable to the wild type in term of vegetative growth (data not shown).

### Wild-Type Micro-Tom Fruit Development and Tissue Morphology

**FIGURE 2:**
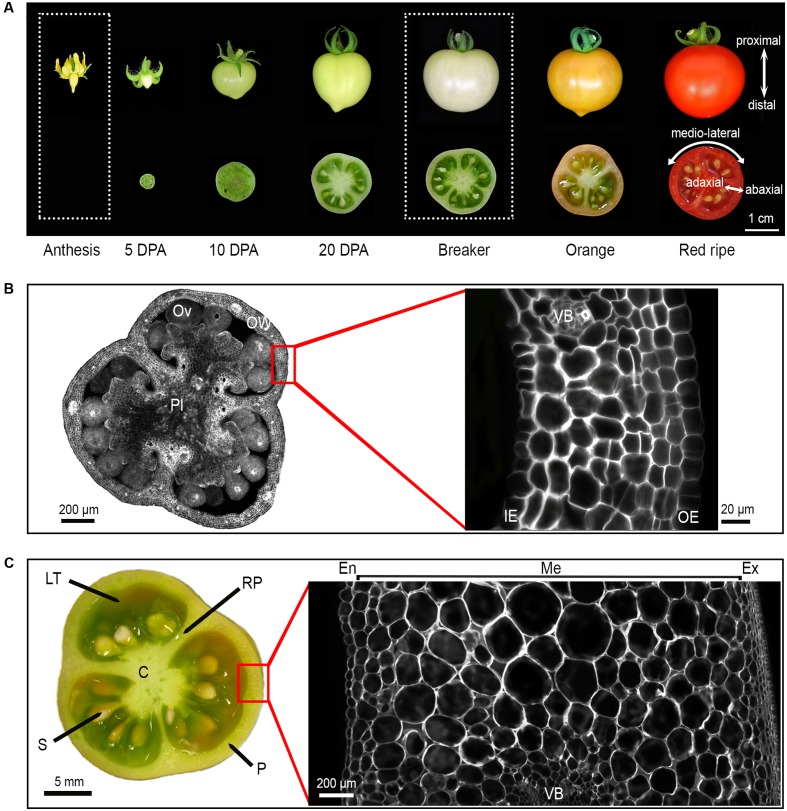
Tomato fruit development and tissue morphology in WT Micro-Tom cultivar. **(A)** Whole fruit and equatorial fruit sections from anthesis to red-ripe stage. The axes of growth: proximal to distal, medio-lateral and abaxial to adaxial are reported. **(B)** Equatorial ovary section and ovary wall section at anthesis stained with calcofluor. **(C)** Equatorial fruit section and pericarp section at breaker stage stained with calcofluor. DPA, Day Post Anthesis; OW, ovary wall; Pl, placenta; Ov, ovule; OE, outer epidermis; IE, inner epidermis, VB, vascular bundle; C, columella; LT, locular tissue; S, seed; RP, radial pericarp; P, pericarp; En, endocarp; Me, mesocarp; Ex, exocarp.

To constitute the reference dataset to which all mutant phenotypic data could be subsequently compared, the tissue morphology of ovary and fruit (breaker stage) from cv. Micro-Tom was thoroughly investigated in WT plants. In Micro-Tom the final fruit size is reached after 30–35 days post anthesis (DPA), at breaker stage (**Figure 2A**). From ovary (1 mm diameter) to breaker stage (2.5 cm diameter), the equatorial fruit diameter increases by more than 2000-fold (**Figures 2B,C**) resulting in a mean fruit weight of 3 g (unrestricted load, 20 fruits per plant) to 5–6 g (controlled load, six fruits per plant). Fruit usually comprises three carpels separated by a radial pericarp (RP; 7% of the fruit area in equatorial section) fused to the central axis called columella (C; 11% of the fruit area) (**Figure 2C** and Supplementary Figure 1A). The pericarp (P) that develops from the ovary wall, and the locular tissue (LT) that differentiates from the placenta are the main fruit tissues, representing, respectively, 30 and 50% of the total fruit area in equatorial section (**Figure 2C** and Supplementary Figure 1A). The number of seeds is proportional to the final fruit weight, ca. 5 to 7 seeds per gram of fruit weight.

Starting from the abaxial (external) to the adaxial (internal) side of the fruit, the pericarp is classically subdivided into: (i) the exocarp, which is constituted by the epidermal cell layer and by several layers of small collenchyma cells; (ii) the mesocarp displaying the smaller cells close to the exocarp and to the endocarp while largest cells are located in the inner mesocarp; and (iii) the endocarp constituted of one epidermal cell layer (**Figures 2A,C** and Supplementary Figure 1B). Vascular bundles, which are constituted by very small phloem cells and by xylem cells, are regularly distributed within the mesocarp (**Figure 2C** and Supplementary Figure 1B). The ovary wall (100–120 μm in thickness) is composed of less than 10 cell layers of uniform small cells (**Figure 2B**). The pericarp (2 mm in final thickness) consists of 14 to 17 layers of cells with a mean area of 0.01 mm^2^, which show considerable size heterogeneity (**Figure 2C**).

### Selected Mutants Exhibit Wide Variations in Fruit Weight, Tissue Morphology, and Cell-Related Traits

**FIGURE 3:**
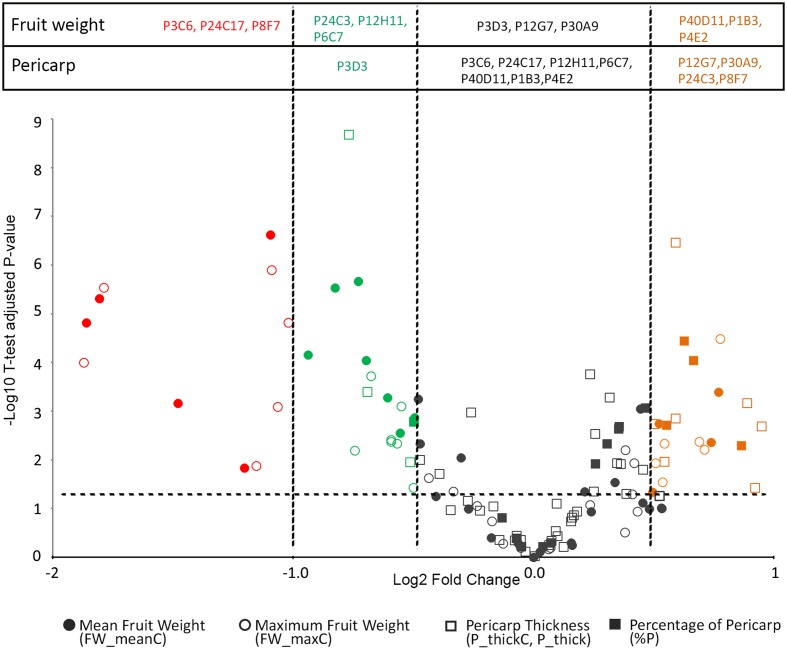
Volcano plot of the variations in fruit weight (FW_meanC and FW_maxC) and pericarp thickness (P_ThickC) or percentage (%P) in the tomato mutants. The Volcano plot combines the magnitude of change plotted on *x*-axis, with the *t*-test significance plotted on *y*-axis indicating the FDR adjusted 0.05 *P*-value threshold. The WT is used as the reference. Log2 Fold change thresholds relative to the WT are indicated using a color code: <–1 (red), >–1 and <–0.5 and (green) and >0.5 (orange).

Final fruit weight was significantly different from that of WT in three large fruit mutants (P4E2, P1B3, and P40D11) and in six small fruit mutants (P12H11, P24C3, P6C7, P3C6, P24C17, and P8F7) (**Figures 1**, **3**). Among these, the P3C6, P24C17, and P8F7 mutants displayed a >2-fold reduction in fruit weight (**Figure 3**). In addition, the P8F7, P24C3, P12G7, and P30A9 mutants produced fruits with a thicker pericarp while the only thin pericarp mutant identified was P3D3 (**Figures 1**, **3**). Noteworthy, although P8F7 and P24C4 were small fruit mutants, their pericarp thickness (P_thick) and percentage of pericarp tissue per fruit (%P) were significantly higher than those of WT (**Figure 3**).

**FIGURE 4:**
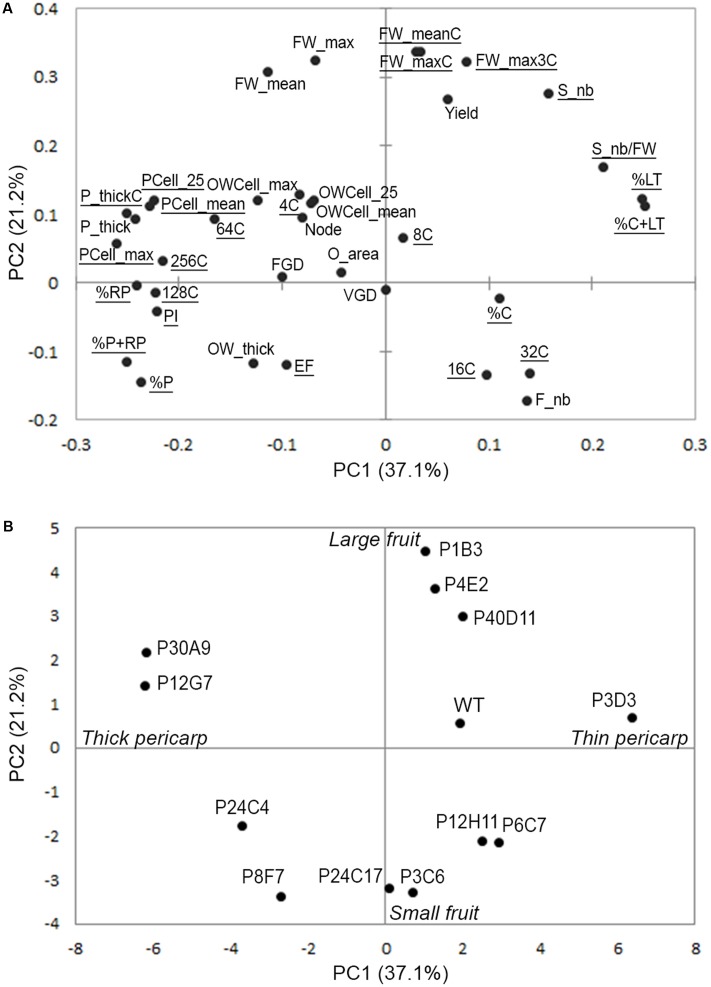
Variability of plant, ovary and fruit traits in the tomato mutants visualized using PCA of 37 traits in 12 mutants and the WT. **(A)** Projection of the 37 phenotypic traits on the first two dimensions PC1 and PC2 explaining 58% of total variance. **(B)** Projection of the mutants and WT on the first two PCs.

To further investigate the contribution of the various plant, fruit, tissue and cell characteristics to the variations in fruit weight and tissue morphology, we performed a principal component analysis (PCA) based on 37 phenotypic traits scored in the 12 mutants and in WT as a reference (Supplementary Table 1). PC1 clearly separated mutants according to pericarp-related traits, which are opposite to locular tissue-related traits in the PCA (**Figures 4A,B**). In contrast, PC2 separated mutants according to final fruit weight (**Figures 4A,B**). Ovary-related traits such as ovary wall thickness and ovary cell area had limited impact on total variation of PC1 and PC2 (**Figure 4A**). However, ovary wall traits together with nuclear ploidy (4C, 8C, and 64C values) of pericarp cells accounted for 14.6% of total variance on PC3 (Supplementary Figure 3). Because the plant-related traits scored (Node, FGD, VGD, Supplementary Table 1) poorly contributed to the total variance, they were excluded from further analyses focused on ovary and fruit traits.

### Fruit Weight and Tissue Morphology Mutants Group in Only Two Clusters Despite Their Diversity

**FIGURE 5:**
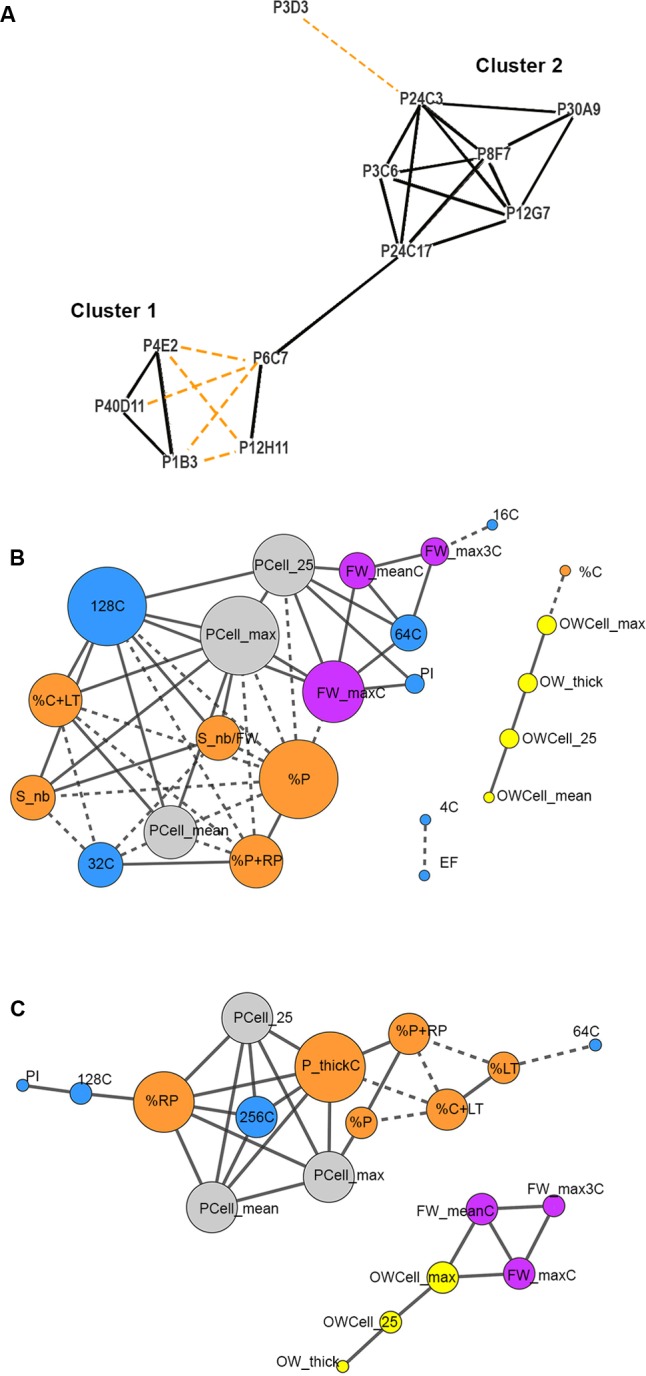
Correlation network analysis of the fruit weight and tissue morphology mutants and of phenotypic traits measured in the tomato mutants. Only significant positive (solid lines) or negative (dashed lines) Spearman rank correlations with **(A)** FDR adjusted *P*-value < 0.01 or **(B,C)** *P*-value < 0.01 are considered in the networks. **(A)** Mutant correlation network based on 24 fruit traits. Mutants whose fruit characteristics have similar trends are clustered together. **(B,C)** Trait correlation networks are represented based on 29 fruit and ovary traits separately for mutant **(B)** cluster 1 and **(C)** cluster 2. A color code is used to distinguish the different subcategories of the phenotypic traits: ovary (yellow), fruit weight (purple), ploidy (blue), cell area (gray), fruit tissue (orange). The size of each node is proportional to the number of correlations with other traits. Phenotypic traits without significant correlations are not reported in the Figure.

To further investigate the contribution of each trait to the variations in fruit weight and tissue morphology, we built correlation networks using Spearman correlations between the 24 fruit traits scored in controlled conditions of fruit production (traits underlined in the PCA shown in **Figure 4A**). The correlation network identified positive and negative relationships between the mutants and, rather surprisingly given the large phenotypic diversity observed in the mutants, clearly separated the mutants in only two distinct major clusters (**Figure 5A**). Cluster 1 included the P4E2, P1B3, and P40D11 large fruit mutants negatively correlated to P6C7 and P12H11 small fruit mutants (**Figures 4B**, **5A**). Cluster 2 included the P3C6, P8F7, P24C17, P30A9, P12G7, P24C3, and P3D3 mutants. Cluster 2 mutants are mainly thick pericarp mutants, except for the P3D3 thin pericarp mutant which is negatively correlated to the P24C3 mutant (**Figures 4B**, **5A**). Cluster 1 encompasses fruit weight variations centered on PC2 while cluster 2 is related to pericarp thickness variations centered on PC1 (**Figure 4A**).

We next built, for each mutant cluster, a correlation network between the parameters describing ovary and fruit characteristics. This allowed us to explore their interrelations at organ, tissue and cellular levels and to analyze their contribution to fruit weight and tissue morphology (**Figures 5B,C**). For both clusters, ovary network was independent from fruit network except that ovary wall thickness and cell size were correlated to columella in cluster 1 and to fruit weight in cluster 2 (**Figures 5B,C**). Thus, fruit weight appears as independent from other fruit characteristics and as related to pre-anthesis ovary development in cluster 2 (**Figure 5C**). For fruit traits, in cluster 1, positive correlations were observed between fruit weight, % of locular tissue and columella, seed number, pericarp cell size and high ploidy (64C and 128C); negative correlations were observed between these traits and lower ploidy values (16C and 32C) and % of pericarp tissues (**Figure 5B**). The positive correlations between fruit pericarp characteristics (proportion in the fruit, thickness and cell size) and high (128C) to extreme (256C) ploidy levels were the main features of cluster 2; negative correlations were observed between these traits and % of locular tissue and columella (**Figure 5C**).

### Common Fruit Developmental Features Characterize Each Cluster of Mutants

**FIGURE 6:**
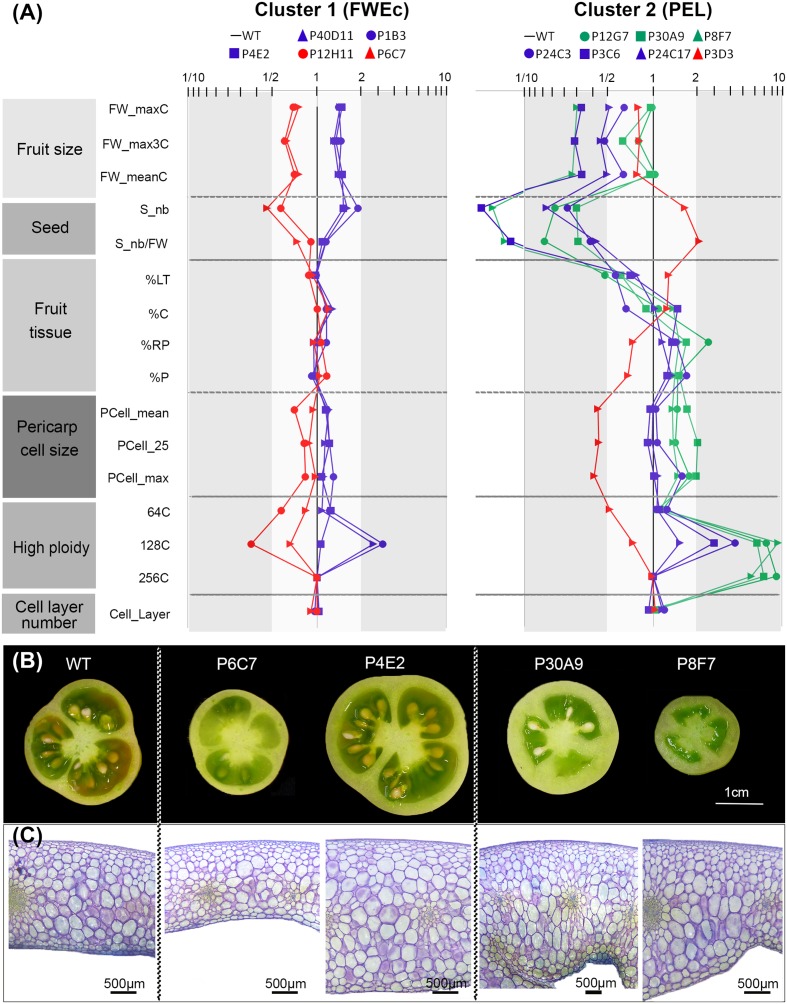
Variation of fruit, tissue and cell-related traits in mutants from the two clusters. **(A)** Fruit trait patterning in cluster 1 associates with fruit weight (FWEc) and in cluster 2 with pericarp elongation (PEL). Value for each trait corresponds to the ratio between the mutant and the WT, plotted in logarithmic scale. The WT is set at 1 to easily identify up and down variations. The color label is used to distinguish ‘low’ (red), ‘high’ (blue), and ‘extreme’ (green) ploidy phenotypes. **(B)** Fresh equatorial sections from breaker stage fruits showing fruit tissue morphology of WT and representative mutants from each cluster. In cluster 1, small fruit mutant (P6C7) and large fruit (P4E2) mutants are shown. In cluster 2, normally sized (P30A9) and small (P8F7) thick-pericarp mutants are shown. **(C)** Pericarp equatorial sections from breaker fruits stained with toluidine blue showing cellular organization in WT and representative mutants in both clusters (same as in **Figure 7B**).

We next focused on main fruit parameters highlighted in the correlation network analysis (**Figures 5B,C**) to investigate more deeply the fruit developmental patterns that characterize the mutants in each cluster. Common features were identified amongst cluster 1 mutants regarding final fruit weight, pericarp cell size and pericarp nuclear ploidy (**Figure 6A**). In this cluster, the relative proportion of the pericarp and of other tissues (locular tissue, radial pericarp, and columella) remained similar to that of the WT, regardless of the final fruit weight (**Figures 6A,B**). Likewise, the seed number remained proportional to fruit weight. Surprisingly, in view of the commonly admitted hypothesis that cell number is a major determinant of fruit weight (Frary et al., 2000), the number of cell layers in the pericarp was barely affected despite the large variation in fruit weight (**Figures 6A,C**). In contrast, the main changes for large fruits were the strong increase in 128C nuclei content and to a lesser extent in 64C nuclei, to which was associated a slight increase in pericarp cell size. The opposite was true for small fruit mutants.

In fruits from cluster 2 mutants, a very different growth pattern was prevailing. In this cluster, fruit weight was either similar to that of the WT or was strongly reduced. However, in contrast to cluster 1, fruit weight reductions were not accompanied by the proportional reduction in all fruit tissues. On the contrary, in all cluster 2 mutants but one (P3D3), the proportion of the pericarp and radial pericarp was substantially increased to reach 40–49% of the fruit tissues (∼30% in WT). Conversely, the proportion of locular tissue was strongly reduced to 27–37% (∼50% in WT), as was the number of seeds (**Figure 6A**). The resulting thick-walled fruits had a bulky and fleshy appearance, reminiscent of processing tomatoes (**Figure 6B**). As already observed for cluster 1 mutants, the number of pericarp cell layers remained largely unaffected. Increased proportion of pericarp tissue in the fruit was associated with increased pericarp thickness and/or pericarp outgrowth in the inner part of the fruit (**Figure 6B**) and with high ploidy values (64C to 256C). Noteworthy, even for the most contrasted P3C6, P8F7, and P24C17 small fruit phenotypes, the pericarp cell area was at least equivalent or higher than that of the WT, while in cluster 1 small fruit phenotypes were associated with smaller pericarp cell area. The thin-pericarp P3D3 mutant, which has the same size as several thick-pericarp mutants displaying high ploidy values, exhibited the opposite trend. Compared to thick-pericarp mutants, the proportions of fruit tissues were inversed (20% of pericarp and 59% of locular tissue) and the fruit had the lowest nuclear ploidy and pericarp cell area values of all the mutants.

### Endoreduplication and Cell Size Are Positively Correlated in Both Clusters

Because endopolyploidy is a major determinant of cell expansion and fruit growth in tomato (Cheniclet et al., 2005; Chevalier et al., 2014), we further examined the pericarp nuclear ploidy levels of the mutants. Mutants were classified according to the proportion of 64C to 256C nuclei into “low” (P3D3, P12H11, P6C7 mutants), “high” (P4E2, P24C17, P40D11, P3C6, P1B3, and P24C4) and “extreme” (P8F7, P12G7, and P30A9) ploidy categories (**Figure 6A** and Supplementary Figure 4). The most striking feature is the large increase in 64C to 256C nuclei in the “extreme” ploidy mutants, and in particular of 128C and 256C nuclei. It is noteworthy to mention that 256C nuclei are not detected in WT and other mutant pericarp cells and that there is a 10-fold increase in 128C nuclei in these mutants compared to the WT (Supplementary Figure 4).

### The Shape of Pericarp Cells Is Markedly Different Depending on the Cluster

**FIGURE 7:**
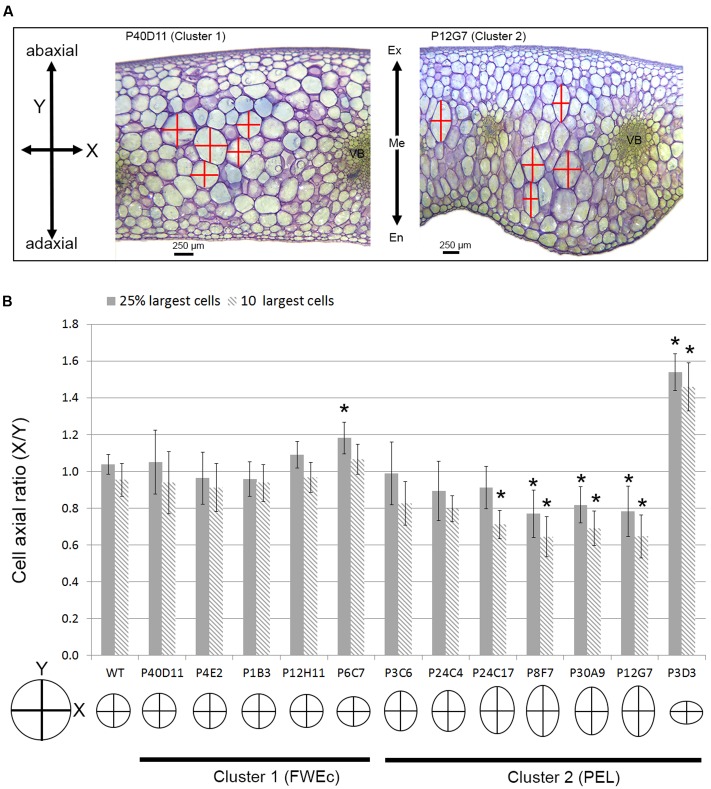
Pericarp cell expansion in fruit weight and tissue morphology mutants. **(A)** Fruit pericarp section stained with toluidine blue for P40D11 (cluster 1) and P12G7 (cluster 2) mutants. In P12G7 mutant, the outgrowth of the pericarp toward the locular cavity of the fruit, forming lobes, is clearly visible as well as the elongated shape of the largest cells. Pericarp cell dimensions along the medio-longitudinal (*X*) and adaxial–abaxial (*Y*) axis used to measure cell shape are indicated. En, endocarp; Me, mesocarp; Ex, exocarp. **(B)** Cell axial ratio (*X*/*Y*) of the 10 and 25% largest cells inside the pericarp for FWEc mutants of cluster 1 and PEL mutants of cluster 2. Asterisks represent significant difference (*t*-test, *P*-value < 0.01) between the mutant and the WT. A representation of cell shape is given for each mutant based on the ratio values obtained for the 10 largest cells.

Even though endopolyploidy is a major factor contributing to fruit tissue growth, and hence to fruit size, additional mechanisms are likely involved in the coordination of fruit growth at organ or tissue levels. As illustrated in fruit pericarp sections (**Figure 6C**), the largest cells appeared more elongated in the thick-pericarp mutants from cluster 2 than in WT or cluster 1 mutants. We therefore evaluated for these cells the *X*/*Y* ratio, where *X* and *Y* describe the cell dimensions according to the two main axes of fruit growth, along the medio-lateral axis (*X*, periclinal growth) and along the abaxial–adaxial axis (*Y*, anticlinal growth) (**Figures 2A**, **7A**). In cluster 1 mutants, the largest cells within the pericarp harbored a round shape except for one mutant (P6C7) where cells were slightly flattened (**Figure 7B**). Strikingly, in cluster 2, several thick-pericarp mutants (P24C17, P8F7, P30A9, and P12G7) showed a significant increase in the cell elongation along the *Y*-axis, which may explain the formation of lobes at the inner face of the pericarp. The thin-pericarp mutant P3D3 displayed the opposite phenotype, with highly flattened cells.

## Discussion

In the recent years, considerable progresses have been made regarding the determinism of the large increase in fruit weight and of fruit shape variations associated with the domestication and subsequent improvement processes in tomato (Lin et al., 2014; Blanca et al., 2015). Analysis of the natural diversity in cultivated tomato germplasm, led to the identification of nine major QTLs and the cloning of six key regulators controlling fruit weight and shape (see Tanksley, 2004; van der Knaap et al., 2014 for review). Remarkably, most QTLs described so far affect floral meristem, ovary carpel development and cell multiplication in the young fruit. Many of these QTLs participate in the same regulatory circuits (Lippman and Tanksley, 2001; Wu et al., 2015; Xu et al., 2015) that control fruit weight and shape, the two traits being not clearly separated. Many questions remain to be addressed. Is there any limit to the increase in fruit weight when the multiplication of carpels as in *lc* and *fas* (Muños et al., 2011; Xu et al., 2015) or the proximal-distal cell multiplication that deforms the fruit as in *sun* and *ovate* (Liu et al., 2002; Xiao et al., 2008) are not considered? Do the late stages of early fruit development (cell expansion stage) contribute to the variations in fruit weight and tissue morphology in tomato and, if so, are these variations controlled by one single or by several developmental modules?

Additional fruit weight and shape QTLs detected in tomato (Grandillo et al., 1999; Gonzalo and van der Knaap, 2008; Huang and van der Knaap, 2011; Rodríguez et al., 2013; Illa-Berenguer et al., 2015) should help address these questions. Many of these QTLs with lower effects are likely modifiers of the major QTLs already identified or may be involved in different fruit growth processes. This would suggest that a larger set of genes governs fruit size. In classical segregating populations used for map-based cloning, the magnitude of the effect of major loci is often overshadowing the smaller effects of minor QTLs. In addition, the reduced genetic diversity available in cultivated tomato and the low number of spontaneous mutants available may not be sufficient to uncover the various circuits regulating fruit growth and patterning (Lin et al., 2014; van der Knaap et al., 2014). Altogether, this may partly explain the low number of fruit weight/shape QTLs cloned to date (<10).

In this context, we reasoned that the new genetic and phenotypic diversity created by EMS saturated mutagenesis may overcome these limitations and help unraveling developmental modules controlling tomato fruit growth (weight and tissue morphology). EMS induces point mutations evenly distributed over the genome, a number of which may be deleterious (Greene et al., 2003). In tomato, EMS mutants already proved to be of great interest for studying various aspects of plant development involved in yield (Park et al., 2014; Xu et al., 2015), leaf development (Berger et al., 2009; Zsögön et al., 2015) or fruit cuticle (Petit et al., 2014, 2016). The mutant collection we developed in the cv. Micro-Tom (Just et al., 2013) is highly mutagenized and, considering the density and impact on protein function of the mutations (Garcia et al., 2016; Petit et al., 2016), 1700 to 3500 EMS mutants are sufficient to reach saturated mutagenesis in tomato (ca. 35 000 genes). Moreover, the genetic background of all the mutant lines from an EMS mutant population is identical, except for the induced mutations, and the phenotypes associated with loss-of-function mutations are revealed in the first mutant generations. For this reason, it is possible to investigate even mild variations across organ, tissue and cellular scales.

Even after excluding hundreds of carpel number and fruit shape mutants from the analysis, our screen led to the successful identification of robust fruit weight and tissue morphology mutants. As expected, these alterations are largely independent from early events affecting ovary and fruit patterning. The cell divisions along the abaxial–adaxial and proximal/distal axes are likely unaffected since we did not observe any variation in fruit shape or in the number of pericarp cell layers. However, increased cell divisions along the medio-lateral axis cannot be excluded, especially in the epidermis of fruits from large fruit mutants. Altogether, our results indicate that alterations in the cell expansion process and hence in cell size are primarily responsible for the variations in fruit weight and tissue morphology observed. An important finding is also the discovery that mutants grouped in two different clusters, affecting either whole fruit growth or more specifically the pericarp. The developmental processes and regulatory circuits affected in our set of selected mutants are therefore likely different from those involved in other fruit weight and tissue morphology variants previously described in tomato (see Tanksley, 2004; van der Knaap et al., 2014 for reviews).

Developmental modules exist from genetic (gene regulatory network or GRNs) to organismal levels and therefore can be studied at nearly every scale of organization (Scarpella et al., 2010; Davila-Velderrain et al., 2014; Zhan et al., 2015). We can consider that the fruit is organized through modules expressing basic behaviors at cellular and tissue levels. For most modules, modular functions are usually carried out by a group of highly interconnected genes involved in a GRN that interact and frequently overlap with other modules (Benítez et al., 2007). In plants, the identification of modules and corresponding GRNs have been to date limited to specific cellular behaviors or developmental processes such as cell fate determination during flower development (Espinosa-Soto et al., 2004; Urbanus et al., 2010; Davila-Velderrain et al., 2014) or leaf development (Ichihashi et al., 2014; González and Inzé, 2015; Horst et al., 2015). The leaf can be represented in a two-dimensional system, thus allowing detailed and combined analysis of genetic interactions, of spatial patterns and of cell type determination and arrangement in the leaf (Scarpella et al., 2010; Ichihashi et al., 2014). However, in contrast to the leaf, the fruit is a bulky organ displaying more complex cellular and tissue patterning. Therefore, the few regulatory networks described for the fruit remain largely incomplete. A starting point to identify such networks is to define the basic set of modules mobilized during fruit growth through a top-down decomposition approach. Based on previous reports, a set of tomato fruit growth modules and their role in the determination fruit weight, fruit shape and tissue morphology can be tentatively identified (**Table 1**). These are early modules controlling either floral meristem cell fate and carpel number or ovary and early fruit growth by cell multiplication (**Table 1**). They include the ‘carpel number’ (CAN), ‘fruit elongation’ (FEL) and ‘fruit weight’ (FWEa and FWEb) modules that may overlap. Interactions of the regulatory networks including the *CLV3-WUSCHEL* circuit and the *SUN* and *OVATE* pathways were proposed to capture most of the fruit shape variations in the cultivated tomato (Rodríguez et al., 2011; Wu et al., 2015; Xu et al., 2015). The FWEa and FWEb modules described so far involve two programs of development governed by the C*NR* and *KLUH* genes, respectively. These modules control the early cell division patterns in the different fruit tissues and are at the origin of variations in fruit weight and, likely, in tissue morphology (see van der Knaap et al., 2014 for review).

**Table 1:** Properties of fruit growth modules.

Module	Cellular events	Morphological roles	*Genes in the GRN*	Reference
CAN^∗^	Cell identity –cell fate	Meristem identity and organization, carpel number, fruit shape	*WUSCHEL (lc) CLAVATA3 (fas) CLE9, HPAT, CLV1*	Muños et al., 2011; Xu et al., 2015
FEL^∗^	Proximal-distal patterning, cell division	Anisotropic ovary and fruit growth (proximal-distal), fruit shape	*OVATE, SUN*	Liu et al., 2002; Xiao et al., 2008; Wu et al., 2015
FWEa^∗^	Cell division	Placenta and columella growth-Ovary and fruit weight	*CNR (fw2.2)*	Frary et al., 2000; Cong et al., 2002
FWEb^∗^	Cell division	Pericarp and radial pericarp growth-Fruit weight	*KLUH (fw3.2)*	Chakrabarti et al., 2013
FWEc^∗^	Isotropic cell expansion	Coordinated tissue growth and fruit weight	*Nd^∗^*	This study
PEL^∗^	Anisotropic cell expansion	Abaxial/adaxial pericarp elongation, pericarp thickness	*Nd^∗^*	This study


*^∗^CAN, carpel number; FEL, fruit elongation; FWE, fruit weight; PEL, pericarp elongation; GRN, gene regulatory network; Nd, not determined.*

Here we identified two additional fruit growth modules, the ‘fruit weight’ FWEc and the ‘pericarp elongation’ (PEL) modules (**Table 1**), which correspond to the two clusters of mutants displaying specific fruit weight and tissue morphology characteristics (**Figures 5**–**7**). Both modules control pericarp cell expansion during early fruit growth, with consequences on either whole fruit (FWEc module; cluster 1 mutants) or only on pericarp tissue (PEL module; cluster 2 mutants). The FWEc module organizes and synchronizes the growth from all fruit tissues. Indeed, the tissue morphology and cell shape isotropy (growth equal in all directions) are preserved whatever the variations in cell size and fruit weight observed in cluster 1 mutants. The PEL module controls pericarp cell elongation along the adaxial–abaxial axis. The resulting cell shape anisotropy (unidirectional growth) is restricted to pericarp and provokes an increase in pericarp thickness and its deformation, as observed for several mutants of cluster 2. Thus, though they are likely interconnected to allow fruit growth and patterning during the cell expansion stage of the fruit, these new fruit growth modules appear to be autonomous, at least to some extent, as the fluctuations in one module do not entail changes in the fruit, tissue and cell characteristics controlled by the second module.

At this stage the modular functions underlying FWEc and PEL modules are unknown and a broad range of gene functions and categories can be involved in the corresponding GRNs. In the FWEc module, factors regulating endoreduplication and cell turgor pressure (Chevalier et al., 2014; Okello et al., 2015) and those involved in non-cell-autonomous signaling pathways are likely candidate. In the PEL module that displays a strong anisotropic growth of the pericarp cells, genes implicated in cell wall and microtubule loading are strong candidates (Verbelen et al., 2001; Baskin and Jensen, 2013).

## Conclusion

Our results support the existence of two distinct developmental modules regulating fruit growth by cell expansion and affecting tomato fruit weight and tissue morphology without altering carpel number and fruit shape. The new insights gained into tomato fruit development and the wealth of data available from this study will considerably contribute to improve the current dynamic models of fruit growth (Baldazzi et al., 2012). Thanks to the recent advances in deep sequencing technologies and to the high quality tomato genomic sequence available (Tomato Genome Consortium, 2012), we recently developed a mapping-by-sequencing strategy readily allowing the identification of causal mutations in the Micro-Tom EMS mutants (Garcia et al., 2016; Petit et al., 2016). Using this strategy, the set of available mutants will therefore help deciphering the genetic network underlying the two modules and shed new light on the poorly known processes controlling fleshy fruit growth.

## Author Contributions

LF and CR designed the research. CM, JJ, LF, and DJ performed experiments. CM, LF, and AM. analyzed data. LF, CM, CR, CC, ML-C, and FG interpreted the data. LF and CR wrote the manuscript.

## Conflict of Interest Statement

The authors declare that the research was conducted in the absence of any commercial or financial relationships that could be construed as a potential conflict of interest.

## Footnotes

1 http://tgrc.ucdavis.edu/

2 https://www.eu-sol.wur.nl/

3 http://biostatflow.org/
