# Supplementary material for: Identification of Two New Mechanisms That Regulate Fruit Growth by Cell Expansion in Tomato
Source: Front Plant Sci. 2017 Jun 12;8:988. doi: 10.3389/fpls.2017.00988 (PMC5467581; doi:10.3389/fpls.2017.00988)
Supplement: Supplementary file 1 [file Table_1.PDF]

Supplementary Material

Identification of two New Mechanisms that Regulate Fruit Growth by Cell Expansion in Tomato

Constance Musseau[1], Daniel Just[1], Joana Jorly[1], Frédéric Gévaudant[1], Annick Moing[1], Christian Chevalier[1], Martine Lemaire-Chamley[1], Christophe Rothan[1,2] and Lucie Fernandez[1,2]*

*** Correspondence:** Lucie Fernandez : lucie.fernandez@inra.fr

Supplementary Table 1. List of the phenotypic traits analyzed in the mutants and WT

Trait name	Trait description	Fruit load[1]	Replicates[2]
Yield	Total fruit weight in total fruit production condition	U	6-10 plants
F_nb	Fruit number per plant in total fruit production condition	U	6-10 plants
FW_max	Maximum fruit weight per plant in total fruit production condition	U	6-10 plants
FW_maxC	Maximum fruit weight per plant in controlled fruit production condition	C	6-20 plants
FW_max3C	Fruit weight average of the 3 larger fruits per plant in controlled fruit production condition	C	6-20 plants
FW_mean	Mean fruit weight per plant in total fruit production condition	U	6-10 plants
FW_meanC	Mean fruit weight per plant in controlled fruit production condition	C	6-20 plants
P_thick	Pericarp thickness in total fruit production condition	U	10-12 fruits
P_thickC	Pericarp thickness in controlled fruit production condition	C	3-6 fruits
%P	Percentage of pericarp relative to total fruit tissue	C	3-5 fruits
%RP	Percentage of radial pericarp (septum) relative to total fruit tissue	C	3-5 fruits
%LT	Percentage of locular tissue (gel) relative to total fruit tissue	C	3-5 fruits
%C	Percentage of columella relative to total fruit tissue	C	3-5 fruits
%P+PR	Percentage of pericarp and radial pericarp relative to total fruit tissue	C	3-5 fruits
%C+LT	Percentage of and columella and locular tissue relative to total fruit tissue	C	3-5 fruits
S_nb	Seed number per fruit	C	5-20 fruits
S_nb/FW	Seed number / fruit weight	C	5-20 fruits
4C (…) 256C	Proportion of pericarp cell nuclei in 4C (…) 256C ploidy level	U and C	12-25 fruits
EF	Endoreduplication factor	U and C	12-25 fruits
PI	Ploidy index	U and C	12-25 fruits
PCell_mean	Mean cell area in the pericarp	U and C	3-6 fruits
PCell_25	Cell area average of the 25% larger cells in the pericarp	U and C	3-6 fruits
PCell_max	Maximum cell area in the pericarp	U and C	3-6 fruits
O_A	Ovary area in equatorial section	C	3-7 ovaries
OW_thick	Ovary wall thickness	C	3-7 ovaries
OWCell_mean	Mean cell area in the ovary wall	C	3-7 ovaries
OWCell_25	Cell area average of the 25% larger cells in the ovary wall	C	3-7 ovaries
OWCell_max	Maximum cell area in the ovary wall	C	3-7 ovaries

Cell_Layer	Number of cell layers in the pericarp from exocarp to endocarp	U and C	4-11 fruits
Cell _shape	X /Y diameter ratio with X=(adaxial-abaxial) and Y=(medio-lateral) directions	U and C	4-7 fruits
FGD	Fruit growth duration (from anthesis to breaker stage)	U and C	20-40 fruits
VGD	Vegetative growth duration (from sowing to 1st flower opening)	U and C	10-20 plants
Node	Node position of the first inflorescence	U	6-10 plants

[1]Controlled (C) or unrestricted (U) fruit load is specified according to the 'Materials and Methods' section

[2]Number of replicates per genotype
