# Supplementary material for: Identification of Two New Mechanisms That Regulate Fruit Growth by Cell Expansion in Tomato
Source: Front Plant Sci. 2017 Jun 12;8:988. doi: 10.3389/fpls.2017.00988 (PMC5467581; doi:10.3389/fpls.2017.00988)

Supplementary Material

Identification of two New Mechanisms that Regulate Fruit Growth by Cell Expansion in Tomato

Constance Musseau[1], Daniel Just[1], Joana Jorly[1], Frédéric Gévaudant[1], Annick Moing[1], Christian Chevalier[1], Martine Lemaire-Chamley[1], Christophe Rothan[1,2] and Lucie Fernandez[1,2]*

*** Correspondence:** Lucie Fernandez : lucie.fernandez@inra.fr

Supplementary Figure 1. Fruit trait measurements. (A) Equatorial section of a breaker fruit analyzed using Tomato Analyzer software. Pericarp (P), radial pericarp (RP), locular tissue (LT) and columella (C) areas are determined according to the whole fruit area (red line). Pericarp area is defined as the area between the red line and the blue line 2. Two supplementary areas defined by the blue lines 3 and 4 are used to determine the remaining fruit tissue areas. (B,C) Equatorial pericarp section at breaker stage stained with (B) toluidine blue and (C) calcofluor allowing pericarp thickness and cell size measurements. Cell segmentation is performed using CellSet software and segmentation is used for automatic cell quantification using Image-Pro PLUS software. P_thick, Pericarp thickness; VB, vascular bundles; En, endocarp; Me, mesocarp; Ex, exocarp; PCell_max, maximum cell area; PCell_25, average area of the 25% largest cells; PCell_mean, mean cell area.

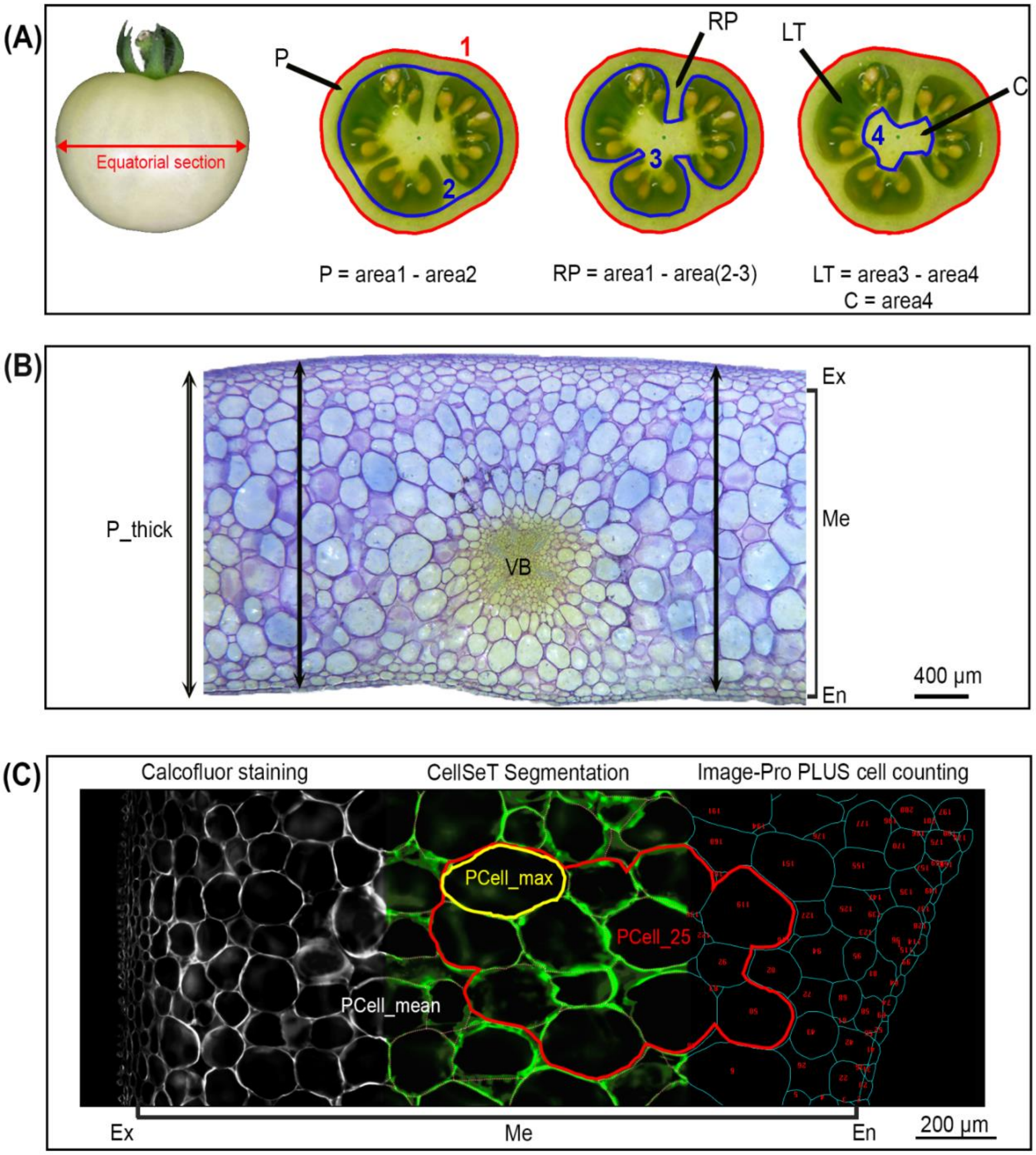

Supplement: Supplementary file 2 [file Image_1.PDF]
