# Supplementary material for: Identification of Two New Mechanisms That Regulate Fruit Growth by Cell Expansion in Tomato
Source: Front Plant Sci. 2017 Jun 12;8:988. doi: 10.3389/fpls.2017.00988 (PMC5467581; doi:10.3389/fpls.2017.00988)

Supplementary Material

Identification of two New Mechanisms that Regulate Fruit Growth by Cell Expansion in Tomato

Constance Musseau[1], Daniel Just[1], Joana Jorly[1], Frédéric Gévaudant[1], Annick Moing[1], Christian Chevalier[1], Martine Lemaire-Chamley[1], Christophe Rothan[1,2] and Lucie Fernandez[1,2]*

*** Correspondence:** Lucie Fernandez : lucie.fernandez@inra.fr

Supplementary Figure 2. Fruit phenotypes of the Micro-Tom EMS mutant collection excluded from the analysis. (A, H) wild-type Micro-Tom (WT) fruit. Examples of (B, C, D, E, F) fruit shape, (G, J) multi-locular and (I) tissue morphology mutants that were excluded from the analysis.

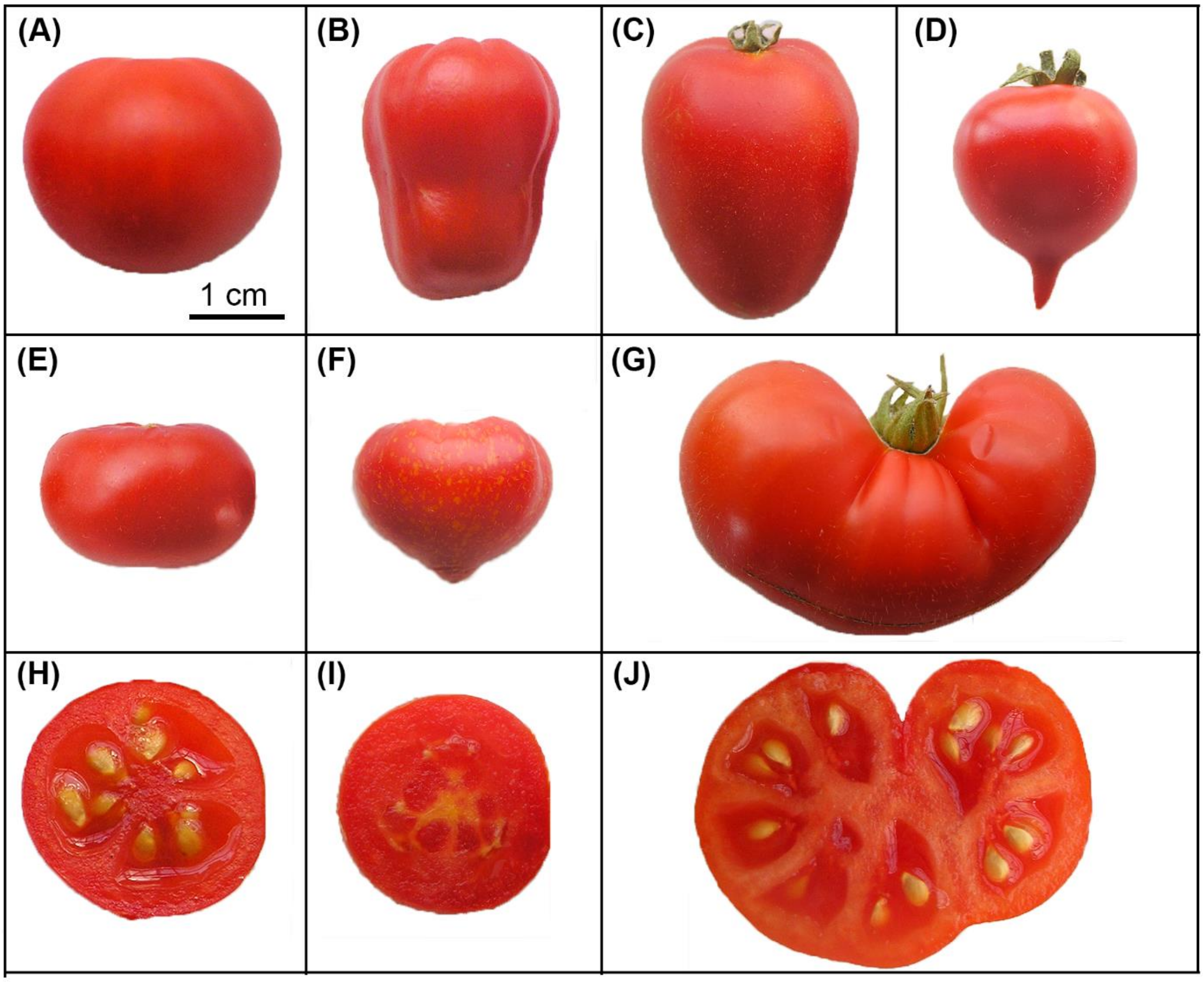

Supplement: Supplementary file 3 [file Image_2.pdf]
