# Supplementary material for: Identification of Two New Mechanisms That Regulate Fruit Growth by Cell Expansion in Tomato
Source: Front Plant Sci. 2017 Jun 12;8:988. doi: 10.3389/fpls.2017.00988 (PMC5467581; doi:10.3389/fpls.2017.00988)

Supplementary Material

Identification of two New Mechanisms that Regulate Fruit Growth by Cell Expansion in Tomato

Constance Musseau[1], Daniel Just[1], Joana Jorly[1], Frédéric Gévaudant[1], Annick Moing[1], Christian Chevalier[1], Martine Lemaire-Chamley[1], Christophe Rothan[1,2] and Lucie Fernandez[1,2]*

*** Correspondence:** Lucie Fernandez : lucie.fernandez@ inra.fr

Supplementary Figure 3. Variability of plant, ovary and fruit traits in the tomato mutants visualized using PCA of 37 traits in 12 mutants and the WT. (a) Projection of the 37 phenotypic traits on the two dimensions PC1 and PC3 explaining 52% of total variance. (b) Projection of the mutants and WT on the PC1 and PC3.

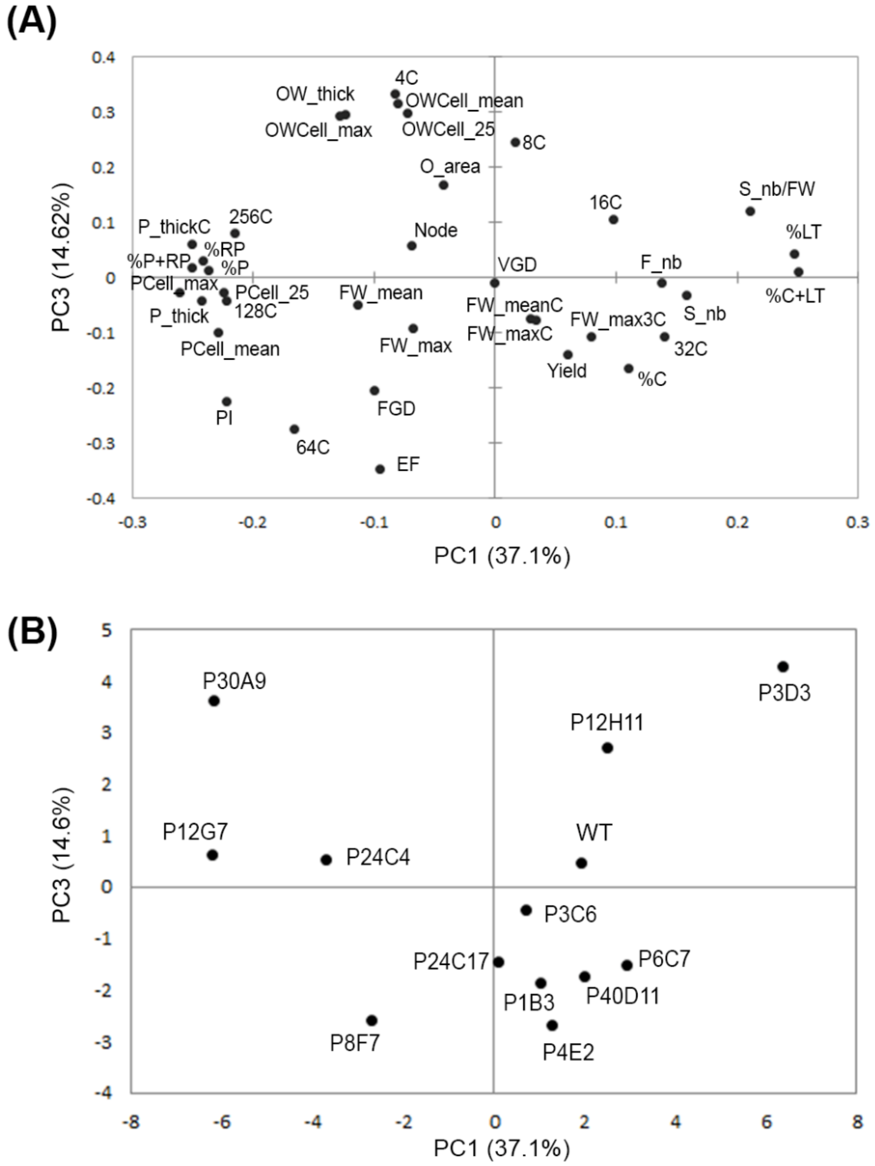

Supplement: Supplementary file 4 [file Image_3.PDF]
