# Supplementary material for: Identification of Two New Mechanisms That Regulate Fruit Growth by Cell Expansion in Tomato
Source: Front Plant Sci. 2017 Jun 12;8:988. doi: 10.3389/fpls.2017.00988 (PMC5467581; doi:10.3389/fpls.2017.00988)

Supplementary Material

Identification of two New Mechanisms that Regulate Fruit Growth by Cell Expansion in Tomato

Constance Musseau[1], Daniel Just[1], Joana Jorly[1], Frédéric Gévaudant[1], Annick Moing[1], Christian Chevalier[1], Martine Lemaire-Chamley[1], Christophe Rothan[1,2] and Lucie Fernandez[1,2]*

*** Correspondence:** Lucie Fernandez : lucie.fernandez@inra.fr

Supplementary Figure 4. Nuclear ploidy profile in the pericarp of tomato fruit in mutants and WT. The mean proportion of nuclei in each ploidy level is plotted and the mutants are classified according to the proportion of high ploidy cells (64C-256C). A color label (same as in Fig. 7A) is used to distinguish low, high and extreme ploidy phenotypes.

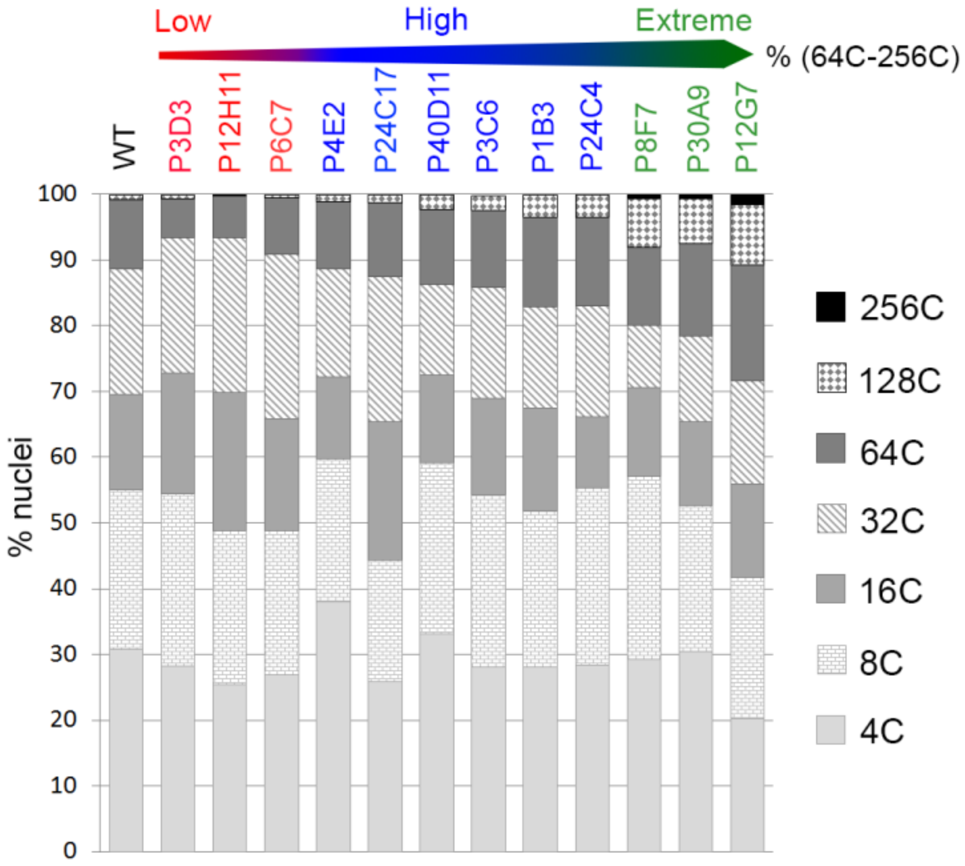

Supplement: Supplementary file 5 [file Image_4.PDF]
